# FLPneXAINet: Federated deep learning and explainable AI for improved pneumonia prediction utilizing GAN-augmented chest X-ray data

**DOI:** 10.1371/journal.pone.0324957

**Published:** 2025-07-17

**Authors:** Shuvo Biswas, Rafid Mostafiz, Mohammad Shorif Uddin, Muhammad Shahin Uddin

**Affiliations:** 1 Department of Information and Communication Technology, Mawlana Bhashani Science and Technology University, Tangail, Bangladesh; 2 Institute of Information Technology, Noakhali Science and Technology University, Noakhali, Bangladesh; 3 Department of Computer Science and Engineering, Jahangirnagar University, Dhaka, Bangladesh; Najran University College of Computer Science and Information Systems, SAUDI ARABIA

## Abstract

Pneumonia, a severe lung infection caused by various viruses, presents significant challenges in diagnosis and treatment due to its similarities with other respiratory conditions. Additionally, the need to protect patient privacy complicates the sharing of sensitive clinical data. This study introduces FLPneXAINet, an effective framework that combines federated learning (FL) with deep learning (DL) and explainable AI (XAI) to securely and accurately predict pneumonia using chest X-ray (CXR) images. We utilized a benchmark dataset from Kaggle, comprising 8,402 CXR images (3,904 normal and 4,498 pneumonia). The dataset was preprocessed and augmented using a cycle-consistent generative adversarial (CycleGAN) network to increase the volume of training data. Three pre-trained DL models named VGG16, NASNetMobile, and MobileNet were employed to extract features from the augmented dataset. Further, four ensemble DL (EDL) models were used to enhance feature extraction. Feature optimization was performed using recursive feature elimination (RFE), analysis of variance (ANOVA), and random forest (RF) to select the most relevant features. These optimized features were then inputted into machine learning (ML) models, including K-nearest neighbor (KNN), naive bayes (NB), support vector machine (SVM), and RF, for pneumonia prediction. The performance of the models was evaluated in a FL environment, with the EDL network achieving the best results: accuracy 97.61%, F1 score 98.36%, recall 98.13%, and precision 98.59%. The framework’s predictions were further validated using two XAI techniques—Local Interpretable Model-Agnostic Explanations (LIME) and Grad-CAM. FLPneXAINet offers a robust solution for healthcare professionals to accurately diagnose pneumonia, ensuring timely treatment while safeguarding patient privacy.

## 1 Introduction

Virus infections (VI) have posed significant risks to human life across the globe, with pneumonia being one of the most common and dangerous types of viral infections [1]. Both bacterial and viral infections can severely damage the lungs [2], leading to symptoms such as fever, cough, discomfort, and difficulty breathing. Each year, pneumonia affects approximately 7.7% of the global population and is a leading cause of mortality among children. A 2019 survey revealed that pneumonia was responsible for the deaths of 740,180 children under the age of 5, accounting for approximately 14% of all deaths in this age group [3]. The highest mortality rates are observed in Central and West Africa and South Asia [4]. Additionally, the risk of pneumonia increases with age, posing a significant threat to individuals over the age of 65 [5]. Chest X-rays (CXRs) are a commonly used method to diagnose pneumonia.

CXRs of the thoracic cavity are typically taken and analyzed by professional radiologists. The presence of white patches in the lungs can indicate infectious regions, and these images may also help identify other complications such as pleural effusions or abscesses. However, interpreting these images requires significant expertise, both in post-X-ray processes and in determining the necessity of radiological imaging [6]. Moreover, this method has its limitations, particularly in elderly patients, where conducting and interpreting high-resolution CXRs can be challenging [7]. Therefore, alternative approaches are needed to address these challenges.

Given that pneumonia shares symptoms with other respiratory illnesses, accurately diagnosing pneumonia based on symptoms alone can be difficult. Automated approaches for pneumonia identification have emerged as crucial tools in this context. Leveraging automated identification techniques through medical data processing, particularly using Deep Learning (DL) and Machine Learning (ML), offers a viable solution [8,9]. However, evaluating DL algorithms requires large amounts of medical data, which presents a challenge for the rapid prediction of pneumonia using deep neural network (DNN) algorithms. A major issue is the limited availability of medical data [10,11]. Due to the rarity of the disease, the small number of available CXR images can lead to overfitting during the training stage of DNN algorithms, resulting in poor performance.

The primary aim of this study is to develop a consistent dataset that enhances the reliability of the proposed architecture. The goal of this manuscript is to create an innovative system for predicting pneumonia in CXR images through integration of the Federated Learning (FL) with CycleGAN [12]. The decentralized FL framework enables collaborative learning across multiple clients without exposing sensitive data. Combining DL techniques that perform image prediction and detection within the FL framework improves the accuracy of the prediction system. This novel combination not only provides precise prediction probabilities but also implements additional security measures [13,14].

Recently, explainable artificial intelligence (XAI)-based algorithms have gained popularity in medical data analysis due to their ability to explain, understand, and visualize ML models used for disease diagnosis [15,16]. Ribeiro et al. [17] introduced a novel XAI algorithm called Local Interpretable Model-agnostic Explanations (LIME) to interpret ML predictions in an understandable manner. Selvaraju et al. [18] proposed another XAI algorithm – the Gradient Weighted Class Activation Mapping (GradCAM) to help understand how ML models make their predictions. Holzinger et al. [19] highlighted the potential of XAI in the future of medical data analysis, allowing medical experts to monitor patient health more effectively. Therefore, the XAI approaches interpret infectious areas in CXR images could assist medical practitioners, especially in rural areas, to better understand the relationship between pneumonia and other respiratory illnesses.

The primary objective of this manuscript is to develop an FL-based ensemble deep learning network (FL-EDLNet) using pre-trained CNN architectures for secure pneumonia prediction. This study also integrates the CycleGAN architecture with EDLNet to address data imbalance issues. The proposed EDLNet aims to assist medical professionals in accurately and securely predicting pneumonia. The main contributions of this work are as follows:

- Utilization of the FL-based ensemble deep learning network (FL-EDLNet) to create a secure environment for pneumonia prediction on CXR images.
- Addressing the imbalanced pneumonia dataset by increasing data through CycleGAN architecture.
- Optimization of the features through recursive feature elimination (RFE), analysis of variance (ANOVA), and random forest (RF) algorithms.
- Application of the XAI techniques to explain the prediction results of the proposed EDLNet.

The remainder of this manuscript is organized as follows: Section 2 reviews the literature, Section 3 explores the methodology, Section 4 presents the results and discussion, and Section 5 concludes with future work.

## 2 Related works

Over the past decade, various researchers have applied DL to precisely recognize respiratory infections and other problems from CXR images. Rajaraman et al. (2017) [20] designed a unique DL-based framework. In lieu of utilizing the whole sample to learn the DL framework, they select an area of interest (AOI) that covers just the lungs. However, there is still a need to modify these approaches for accurate pneumonia detection. In a different study, Siddiqi et al. [21] developed an eighteen-layer DNN architecture and trained their model using the paediatric CXR dataset. This study demonstrated high classification accuracy (94.39%), specificity (86%), and sensitivity (99%). In [22], the authors developed a DNN approach named CovXNet to diagnose bacterial pneumonia, viral pneumonia, and COVID-19. Their dataset includes 1,583 normal cases, 1493 non-COVID-19 pneumonia samples, 2980 cases of bacterial pneumonia on X-rays, and 305 cases of COVID-19 on CXR scans from various patients. Their framework’s performance has specificity, accuracy, precision, recall, AUC, and Fl score of 89.1%, 90.2%, 90.8%, 89.9%, 91.1%, and 90.4%, respectively. In order to classify CXR pictures into the three categories of TB, viral pneumonia, and bacterial pneumonia, Verma et al., 2020 [23] presented a four-layer customized DNN architecture. They used image augmentation techniques to mitigate the overfitting issue. They recorded a high level of accuracy. However, their publication lacks information on the experimental examination’s specifics. In [24], the authors proposed a specially designed DNN architecture with four convolutional layers for feature extraction, two dense layers, and then the output layer for final prediction. They conducted data analysis and tested the framework with a variety of sample sizes. Over the entire dataset of different sizes, they reported an average accuracy of 93.01%. However, other crucial performance indicators like specificity, recall, or sensitivity value were not present when the system was analyzed. As a result, it is not possible to evaluate the framework’s accuracy using other performance metrics. Jain et al. [25] reported DNN frameworks for dividing pneumonia from normal in CXR images. The number of used parameters, convolutional layers, and hyperparameters varied among these models. The first two frameworks containing 3 Convolution layers offer 92.31% and 85.26% of accuracy, respectively, while the accuracy of the pre-trained networks (InceptionV3, VGG16, ResNet50, and VGG19) is 70.99%, 87.28%, 77.56%, and 88.46%. The models under discussion primarily focus on the recall metric as a performance indicator to reduce the false negative (FN) score. The second framework had the best outcomes, with a 98% recall rate. However, these models require fine-tuning each parameter and hyperparameter to increase the classification accuracy. Saraiva et al. [26] created a multilayer perceptron (MLP) and CNN algorithm to detect the presence of pneumonia in a CXR image. They evaluated the diagnostic accuracy of the two networks for pneumonia on 5863 X-ray pictures. CNN and multilayer perceptron models showed the best accuracy at 94.40% and 92.16%, respectively. A study by Jaiswal et al. [27] utilized a mask recurrent convolutional neural network (Mask RNN) to find pneumonia symptoms in sample data. They had already trained their network on COCO weights to determine DL characteristics. They used image augmentation during model training for generalization in recognizing the presence of the virus. They train their mask RCNN using the publicly accessible chest X-ray dataset from RSNA, an optimal subset of the original 112,000 CXR sample data. In [28], the authors discussed some crucial requirements for accurately and rapidly identifying pneumonia, especially in cases involving fragile young patients. They presented an optimized DL-based framework named MobileNet, and they achieved a significant combined accuracy of 97.09% with 96% specificity, 97% precision, 97% F measure, and 98% recall. The framework’s accuracy, as exhibited through its faster training period and low computing complexity, as well as its massive achievement in quick pneumonia prediction.

The majority of articles on pneumonia prediction in the aforementioned works restrict their focus to the implementation of traditional DNN approaches. A limited number of papers have focused on ensemble DL [29,30] to improve the prediction results of their designed architectures, and some proposed architectures have been customized for particular objectives [25,31]. The majority of articles have implemented conventional image augmentation methods to enhance the volume of the training data [32,33]. But these augmentation methods have pitfalls, because they may not tackle all feasible modifications that a framework might face in practical circumstances. As a result, researchers have searched for sophisticated augmentation methods, such as GAN, to produce more realistic and diverse augmented images [34, 35]. However, a limited number of manuscripts have focused on data privacy issues [36]. To our exploration, there is no prior work that has presented strategies to predict pneumonia utilizing FL. Overall, there are numerous DNN frameworks for pneumonia prediction; attention-based techniques enhance the framework’s prediction ability, multi-scale feature extraction techniques assist in fine-tuning the framework; and feature optimization techniques improve the framework’s performance through selecting the optimal features. In light of the above issues, we propose a multi-scale feature extractor that retrieves features using two pre-trained CNN algorithms in parallel along with a feature optimizer. Finally, we have applied an ensemble ML classifier to predict pneumonia. Our framework is unique, particularly from current techniques, in that it incorporates several feature optimizers, reduces computational complexity, and requires limited memory.

In summary, this manuscript has offered a reliable and secure system to tackle the issues previously stated. This system eliminates the need for information sharing among clients by combining FL and DL algorithms. We used CycleGAN to create synthetic data because the available dataset on CXR images of pneumonia patients was small. Ultimately, we apply XAI algorithms to elucidate the predicted outcomes of DL models Table 1 highlights the method, strengths, and weaknesses of the prior work.

**Table 1:** Summary of some published articles on pneumonia detection.

Ref.	Method	Strength	Limitation
Siddiqi et al., 2019 [21]	18-layer deep CNN architecture for prediction	Fast training process even for highly complex CNNs	Model training from the ImageNet database may include negative data distribution; when the model is pre-trained on a sample not similar to chest data
Li et al., 2020 [22]	3D deep CNN model, named CovXNet, to predict pneumonia based on ResNet50	Transfer learning-based detection of pneumonia exhibits a very remarkable outcome	Only employs frontal chest data, which may not always be sufficient for pneumonia identification
Verma et al., 2020 [23]	4-layer DCNN architecture to detect pneumonia efficiently	Exhibits a very high accuracy of 99.01%	Lacks of information on the specifics of the experimental examination
Stephen et al., 2019 [24]	4 layered-CNN utilized for prediction of pneumonia	Simple CNN model with 93% accuracy on validation	Manuscript does not indicate significant metrics like precision, F-measure, and recall
Jain et al., 2020 [25]	Use of a highly understandable beneficial approach to pneumonia diagnosis	Research demonstrates that it is possible to obtain superior results through their method	Although the framework is understandable, the precise reason behind the framework’s prediction is obscure
Saraiva et al., 2019 [26]	Creation of a deep ensemble pneumonia detection network using CNN and multilayer perceptron (MLP)	Work shows that it is possible to evaluate the generalization capacity through cross validation	Lack of data security
Jaiswal et al., 2019 [27]	Creation of a DCNN model and pre-trained on COCO weights using Mask RCNN	Obtains robustness through complex changes to the training time	Lack of explainability and interpretability of the framework’s prediction

## 3 Methods and methodology

**Fig 1:**
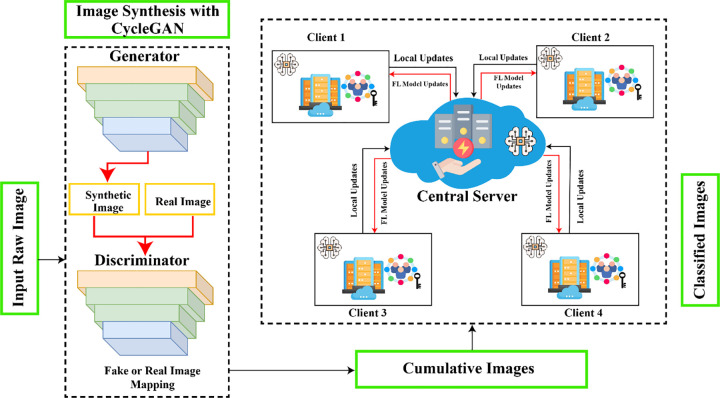
Our system architecture.

The offered framework has two main parts: (i) image synthesis with CycleGAN and (ii) evaluating ensemble DL models in an FL environment. First, we evaluated several DL algorithms separately, and then we merged our DL algorithms on a FL platform to examine their efficacy in both scenarios. Because of the insufficient dataset, the initial stage entailed producing augmented pictures using CycleGAN. Finally, we conducted an optimized ensemble DL (EDL) model with an ML classifier for pneumonia prediction. The optimized EDL model consists of three parts: (i) feature extraction using multiple pre-trained CNN algorithms; (ii) feature selection using several feature selection methods; and (iii) final prediction using several ML classifiers. However, this section presented a broad elucidation of the proposed system, step by step. Fig 1 illustrates the offered approach structure.

### 3.1 Dataset description

Identifying pneumonia from the normal CXR images is a type of binary classification challenge. To build an accurate prediction system, it is crucial to form an optimal training set. The collected dataset D was expressed as follows:


$$D=D_{NORMAL}\cup \;D_{PNEUMONIA}$$

Here D_NORMAL_ represents the set of negative sample data that only contains normal CXR images and

D_PNEUMONIA_ represents the set of positive data that only includes pneumonia CXR images. The symbol ∪ indicates the “union” of these two sets.

**Fig 2:**
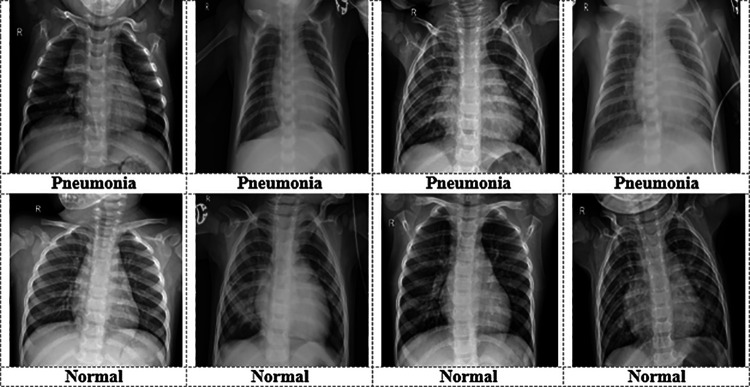
Some sample data from the CXR dataset.

**Fig 3:**
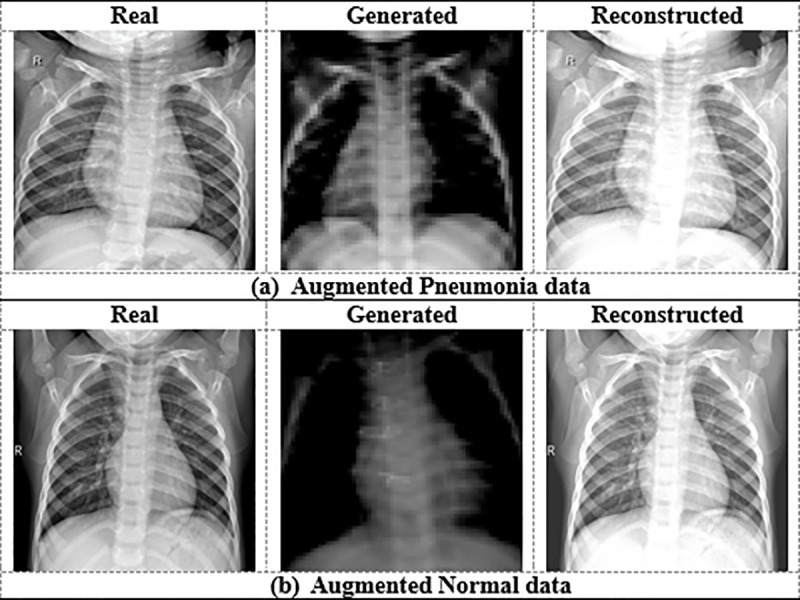
Some augmented samples through CycleGAN.

A benchmark dataset of chest X-ray images (CXR) [37] is utilized to conduct this experiment. The working dataset consists of 5856 anterior-posterior CXR images, including 1583 normal images and 4273 pneumonia images. These pictures were carefully chosen from historical records of pediatric patients aged 1–5 years old [38] at Guangzhou Women and Children’s Medical Center in Guangzhou. The patients underwent CXR imaging as part of their regular medical treatment. We first checked all chest (anterior-posterior) radiographs for quality, eliminating any unreadable or low-quality scans before exploring the CXR pictures. Before approving their AI algorithm’s evaluation, two medical experts further examined the detected pictures. Lastly, a third expert verified the assessment set to ensure there were no grading issues. We partitioned the collected CXR dataset into 80% (4684 samples) for training objectives and 20% (1172 samples) for testing. We collected the CXR dataset from a publicly available repository, which only included JPEG pictures. Fig 2 represents some examples of the experimental data. Because of their accumulation from different patients, the images exhibit a variety of sizes and formats, making them unsuitable for statistical analysis. To solve this problem, we have scaled all the samples to align with the precise algorithm specifications. However, in the CXR dataset, the normal sample size is significantly smaller than that of the pneumonia samples. We apply a CycleGAN image augmentation method (briefly described in Section 3.2) to overcome this data unbalancing situation and produce augmented samples. After GAN-augmentation, the size of the train set increased from 4684 to 7230, with 3643 images of pneumonia and 3587 images of normal. Finally, we conducted this experiment using a total of 8402 CXR images. Fig 3(a) represents three samples of GAN-augmented pneumonia data, while Fig 3(b) represents three samples of GAN-augmented normal data. Table 2 shows the CXR data distribution before and after GAN-augmentation.

**Table 2:** CXR data distribution before and after GAN-augmentation.

		Train set (80%)		
Dataset	Class	Original	After GAN-augmentation	Test set (20%)
	PNEUMONIA	3418	3643	855
CXR	NORMAL	1266	3587	317
	Total	4684	7230	1172

### 3.2 Data augmentation with CycleGAN

**Fig 4:**
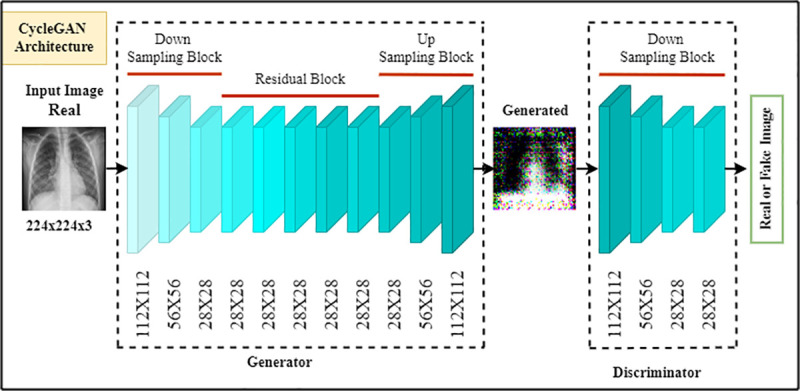
CycleGAN approach with discriminator and generator.

CycleGAN (Cycle Consistent Generative Adversarial Network) makes fake samples from real images without needing a coupled training set. Three crucial components—a discriminator, a generator, and two neural networks—are utilized in this mechanism to extract new samples simultaneously. The proposed scheme illustrates the bidirectional execution of data translation. However, the discriminator’s function is to judge the quality of the sample produced by the translation stage between the pneumonia and healthy X-ray image domains, and vice versa. This article employs this mechanism to generate synthetic samples, allowing the proposed EDL model to function effectively with an unpaired sample. CycleGAN is suitable because the dataset size in the field of pneumonia prediction is limited, and collecting the necessary data is difficult. Fig 4 [39] depicts the proposed scheme of CycleGAN.

### 3.3 Building an optimized ensemble deep learning network (EDLNet)

**Fig 5:**
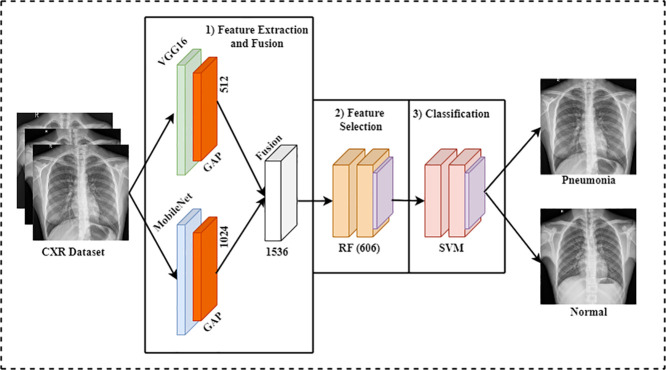
Schematic of the optimized EDLNet for pneumonia prediction. GAP indicates GlobalAveragePooling Layer, RF indicates Random Forest feature optimizer and SVM indicates Support Vector Machine algorithm for Pneumonia classification.

An optimized ensemble deep learning network (EDLNet) is constructed in this article that accurately identifies pneumonia from the CXR dataset compared to the traditional DL network. Fig 5 illustrates the EDLNet’s construction process. The construction process primarily consists of three stages: (i) feature extraction and fusion, (ii) feature selection, and (iii) final prediction. In the feature extraction step, we trained two different CNN algorithms (VGG16 and MobileNet) to function as the feature extractor, extracting high-resolution features from the GAN-augmented dataset. We then use a concatenation layer to fuse the retrieved characteristics from the pre-trained CNN algorithms, forming a blended feature set for more informative data. In the feature selection stage, we then applied three feature selection methods (analysis of variance (ANOVA), random forest (RF), and recursive feature elimination (RFE)) to choose the best subset of features from the retrieved features. Finally, we feed the selected best subset into several ML classifiers, including naive bayes (NB), support vector machine (SVM), random forest (RF), and K-nearest neighbour (KNN) to predict pneumonia. The construction process of the optimized ensemble DL model is outlined in the next sub-sections sequentially. Lastly, the pseudocode for the EDL model is shown in **Algorithm 1**.

**Algorithm 1** Pseudo code of the EDL model.

***Input****: Original chest X-ray data set D*_*CXR*_ *and its class labels: D*_*PNEUMONIA*_ *and D*_*NORMAL*_

***Phase I:*** *Crop black edges from D*_*CXR*_ *→ D*_*Crop*_

***Phase II: Select the pre-trained DL (PDL) algorithms for feature extraction***

* Load the traditional PDL algorithm: M*_*VGG16*_*, M*_*NASNetMobile*_ *and M*_*MobileNet*_;

*Pre-train on them and make several combined models to choose the best feature extractor one:*

*M*_*BestExtractor*_ *= Max [Best Extractor (M*_*VGG16*_ *, M*_*NASNetMobile*_ *, M*_*MobileNet*_ *, M*_*VGG16+NASNetMobile*_ *, M*_*NASNetMobile+MobileNet*_ *, M*_*MobileNet+ VGG16*_ *, M*_*VGG16+NASNetMobile+MobileNet*_ *)];*

***Phase III: Choose the best feature optimizer***

* Load the feature optimizer: O*_*ANOVA*_*, O*_*RF*_ *and O*_*RFE*_;

*Train them on the extracted feature set to select the best optimizer:*

* O*_*BestOptimizer*_ *= Max [Best Optimizer (O*_*ANOVA*_*, O*_*RF*_*, O*_*RFE*_*)];*

***Phase IV: Train and Test ML classifier (MLC) on the Optimized feature set (OFS)***

* Split optimized feature set D*_*Opt*_ *into train data and test data: D*_*Opt*_ *→* $\left\{D_{opt}^{train\left(80\;\%\right)},\;D_{opt}^{test\left(20\;\%\right)}\right\}$

* The training set* $D_{opt}^{train}$ *for class labels* $L_{opt}^{train}$

* The testing set* $D_{opt}^{test}$ *for class labels* $L_{opt}^{test}$

* Load four ML classifiers, C*_*i*_*: C*_*KNN*_*, C*_*SVM*_*, C*_*RF*_ *and C*_*NB*_;

*Train and test them on the OFS to select the best classifier:*

***for*** *i = 1:4*

 Training Accuracy: Accuracy (i) = fit ($D_{opt}^{train,\;i},\;L_{opt}^{train,\;i})$;

 Prediction: Predict(i) = pred ($D_{opt}^{test,\;i}$);

 Test Confusion matrix: ConfTest = con_matrix (Predict(i), $L_{opt}^{test,\;i}$);

 Evaluate Indicators: recall, accuracy, F measure, precision, and AUC.

***end***

***Output: The top EDL framework with performance indicators.***

#### 3.3.1 Feature extraction.

Medical radiography images, especially CXRs, intrinsically contain valuable information. Unique characteristics, such as patterns, textures, and forms, are critical for pneumonia prediction. To successfully retrieve these unique characteristics in CXR images, we applied two powerful DCNN algorithms, namely MobileNet and VGG16, as the DL feature extractor. These two extractors extract the most valuable feature maps from the CyclGAN-augmented data in parallel.

**(i) MobileNet.** MobileNet is a lightweight, deep CNN (DCNN) version that is commonly applied in embedded strategies for diagnostic-based systems. The DC (depth-wise convolutions) allow this DCNN to have fewer DL parameters, which reduces the computational complexity. The operation of this network is first the DC, follwed by the PC (point-wise convolution) [40]. The convolution process of the MobileNet is expressed by the following equation 1:


$$T*K=\sum_{j=1}^{c_{j}}T_{j}*\;K$$ (1)

Here T indicates the input tensor, K is the kernel, and Tj denotes the tensor’s j-th component, respectively, and * indicates the CO (convolution operation). However, after performing the component-wise product and moving the K (kernel) over the T (input tensor) in the CL (convolutional layer), the final result of the CO is calculated by combining the two. However, this experiment applies the MobileNet DCNN model as the first feature extractor that retrieves 1024 higher level features.

**(ii) VGG16.** The structure of VGG16 DCNN was designed by Simonyan [41] and presented in the ILSVRC (2014) competition, where it recorded 92.7 percent accuracy on the large database (ImageNet). This DCNN model has a total of 16 deep NN layers, containing thirteen convolution (CNV) layers, five pooling (POL) layers and three fully connected (FLC) layers. The dimension of the CNV filter in the CNV layers is 3 × 3 with a fixed stride size of 1. In the POL layers, the filter’s dimension is 2 × 2, with the step fixed at 2. Each CNV layer uses an activation function (ACF), also known as the rectified linear unit (ReLU). This DCNN model allows a 224 × 224-pixel image with an RGB channel as input. The first portion has two CNV layers, accompanied by a POL layer. These CNV layers have 64 filters with 224 x 224 pixels. However, this experiment employs the VGG16 DCNN model as an additional feature extractor, retrieving 512 higher-level features.

#### 3.3.2 Feature fusion and selection.

In this section, the retrieved information sets from the above deep feature extractors are combined by applying a concatenation layer to make a fused set of size 1×(a + b), where a = 1024 and b = 512 are the number of extracted features from MobileNet and VGG16 models. Thus, this feature fusion technique extracts a total of 1536 DL features. With the combined power of MobileNet and VGG16, our ensemble deep learning (EDL) is capable of retrieving more DL characteristics than a single network from the input image. As a result, this EDL model paves the way for reliable and accurate pneumonia prediction by extracting more features. Table 3 provides a summary of the output of each layer of the EDLNet. We retrieved this table during the simulation of the EDLNet’s pneumonia/normal prediction model. That’s why the final dense layer contains two neurons.

**Table 3:** Summary of the output of each layer of the EDLNet.

Layer (type)	Output Shape	Param #	Connected to
input_1	(224, 224, 3)	0	
mobilenet_1.00_224	(7, 7, 1024)	3228864	input_1[0][0]
vgg16	(7, 7, 512)	1471468	input_1[0][0]
GlobalAvgPool2D	(1024)	0	mobilenet_1.00_224[0][0]
GlobalAvgPool2D_1	(512)	0	vgg16[0][0]
concatenate	(1536)	0	GlobalAvgPool2D[0][0] GlobalAvgPool2D_1[0][0]
dropout	(1536)	0	concatenate[0][0]
batchNormalization	(1536)	6144	dropout[0][0]
dense	(128)	196736	batchNormalization[0][0]
dropout_1	(128)	0	dense[0][0]
batchNormalization_1	(128)	512	dropout_1[0][0]
dense_1	(2)	258	batchNormalization_1[0][0]
Total params: 18,147,202 Trainable params: 18,121,986 Non trainable params: 25,216			

The combination of multiple DCNN models makes the ensemble architecture complex, and the size of the retrieved fused set (RFS) is particularly large. Directly using the RFS to train the ML predictors requires high computation power and a large amount of memory. As a novelty, this paper presents the optimization challenge of selecting optimal features from the fused set. Thus, a novel optimization algorithm makes the ensemble architecture’s training time faster as well as reducing computational demand compared to other systems in the previous works. For this, three feature optimization techniques (analysis of variance (ANOVA) [42], recursive feature elimination (RFE) [43] and random forest (RF) [44]) are used to address the aforementioned problem. Among these three optimizers, random forest (RF) is considered the best feature optimizer based on its performance. Below is a brief explanation of this algorithm.

A popular supervised machine learning (SML) technique for both regression and prediction difficulties is RF [44]. It depends on multiple ensemble-functioning DT (decision tree) algorithms. The RF algorithm’s final result is determined by majority voting. The RF feature optimizer’s main idea is that each DT computes the value of a feature based on its capacity to boost the node’s purity. While expanding the DTs, it randomly selects the optimal features from the fused set. The better the enhancement in the node’s purity, the greater the impact of the characteristics. The calculated feature values from each DT are then averaged and, subsequently, normalized to 1.0. Finally, the RF computes a total sum of 1.0 feature values.

#### 3.3.3 Classification.

We then feed the selected optimal feature set from the RF algorithm into several ML predictors for pneumonia prediction. In this work, we applied five popular ML predictors, namely KNN [45], SVM [46], RF [44] and NB [47], for accurate pneumonia prediction. Based on its performance, SVM is considered the best ML predictor among these. A brief explanation of this algorithm is described below:

Support Vector Machine (SVM) [46] is a popular SML algorithm for regression and prediction. SVM predicts features by converting each optimized feature into an intricate feature set. Separating the feature into two labels, SVM generates a hyperplane (HPL). The optimal HPL is obtained by the SVM for the linear feature by reducing the marginal distance (MD) between two labels, like {pos (+1) and neg (−1)} and reducing the generalization risks. The optimized fused set defined by F={(F1, y_1_), (F_2_, y_2_),.., (F_n_, y_n_)} represents the training sample with pairing pos (+) or neg (-) labels y_i_, here *y*_*i*_*∊ {+1 or −1}*. However, the distinct HPL is created utilizing formula 6 by computing the maximization distance of *M*^*T*^ *F*_*k*_* + a = −1* for y_k_ = prediction neg (-) and *M*^*T*^ *F*_*k*_* + a = +1* for *y*_*k*_ = prediction pos (+). Equations 2 and 3 determine the prediction accuracy of the SVM.


$$M^{T}F_{k}+a=0$$ (2)

$$y_{k}.M^{T}F_{k}+a\geq 1$$ (3)

Here, *f(*$\hat{M}$, $\hat{Fi}$) represents the optimization function such that the score of $\frac{2}{\left|M\right|}$ must be maximum. Equation 4 provides the formula for the HPL with a magnitude of MD.


$$(\hat{M},\;\hat{Fi})=min\frac{\left|M\right|}{2}+C_{k}.\sum_{k=1}^{l}\mu_{i}$$ (4)

Here, μ is the kernel parameter and *C* is the regularization parameter.

### 3.4 Federated learning with deep learning model for pneumonia prediction

As a novelty, an ensemble federated learning (EFL) system utilized in this paper is performed utilizing the Flower FL architecture. Then, we trained three traditional deep CNN (DCNN) algorithms (MobileNet, VGG16, and NASNetMobile) and the customized EDLNet on the FL environment for the purpose of data security and an improved result. Table 4 shows the best hyperparameters and their values utilized to train these models. The central server or platform then sets up the global DCNN algorithm. Each DCNN algorithm awaits client data. The local platform’s clients attach to the global platform, access the global DCNN algorithm, and then initiate the local model training utilizing local information. The clients transfer their modifications to the global DCNN algorithm without sharing their private sample data. Thus, when the central platform has collected all types of modifications, it applies the FedAvg technique (see equation 5) [48] to merge them.

**Table 4:** Parameter settings for training of FL-based DL models.

Hyperparameters	Values
Optimizer	Adam
Metrics	Sparse_categorical_crossentropy
Loss Function	Sparse_categorical_crossentropy
Epochs	20
Learning Rate	0.001
Batch Size	32


$$p_{T+1}^{m}=\frac{1}{c_{k}}\;\sum_{k=1}^{c_{k}}\omega_{k}*p_{T}^{k}$$ (5)

Here $p_{T+1}^{m}$ denotes the global DCNN algorithm modification at time period (T + 1), *c*_*k*_ represents the total clients used in the averaging process, ω_*k*_ indicates the weights applied to each client to perform averaging, and $p_{T}^{k}$ represents the local algorithm component on platform *k* at time *T*.

### 3.5 Explainable artificial intelligence

Explainable artificial intelligence (XAI)-based algorithms are becoming developed day by day, especially in domains like medical image analysis where quick decisions are crucial [49,50]. We then explain the prediction results of the DCNN algorithm to medical experts to enhance their understanding and readability, enabling them to make accurate and timely predictions of pneumonia and other diseases [51,52]. This experiment incorporates two well-known XAI algorithms, GradCAM and LIME.

#### 3.5.1 Local interpretable model-agnostic explanations (LIME).

For an understandable explanation of the root visualization of a sample being interpreted (*x ∈* ^*k*^) utilizing LIME [17], a binary feature map (*x∈{0, 1}*^*k*^) indicating the “absence” or “presence” of a constant region of super-pixel was utilized. For the proposed system *m ∈ M* having a range of *{0, 1}*^*k*^ to represent the important features, *m* performed over the presence or absence of the explainable features. It was found that each feature of *m ∈ M* was insufficient for understanding the interpretation, so *Ω(m)* was imposed to compute the complexity of the interpretation. Equation 6 provides LIME’s feature explanation formula.


$$\xi (x)=arg_{m\in M}\;maxℑ(f,g,\pi_{x})+\Omega \left(m\right)$$ (6)

Here For our model *f(x):* ℝ^*k*^ *→* ℝ is the probability that *x* belongs to one of two classes, and *π*_*x*_*(y)* is the proximity indicator of a sample from range *y* to *x. ℑ(f, m, π*_*x*_*)* is the fidelity function applied to measure how unfaithful *m* is in similar to *f* in the locality represented by *π*_*x*_. To maximize feature explanation, the fidelity function should be reduced by keeping the value of *Ω(m)* as low as possible.

#### 3.5.2 Gradient-weighted class activation mapping (GradCAM).

GradCAM [18] computes the gradient score of a differentiable result, like a class value, with respect to the DL characteristics of the last CONV layer. Medical image analysis widely uses GradCAM, which is also applicable for image segmentation. However, the softmax layer of the DCNN architecture predicts a value for every pixel and class to assist in the segmentation process. In mathematical terms, for a target class *c* having *P* pixels and *an Am* activation map, GradCAM mapping followed by a ReLU activation function may be defined by equation 7 [53].


$$M_{GradCAM}^{c}=\;ReLU\left(\sum_{m}\varphi_{m}^{c}A^{m}\right)$$ (7)

Here $\varphi_{m}^{c}\;$ is the neuron weights that contains the importance of activation map *m* for a specific class *c* and this weight can be defined by equation 8.


$$\varphi_{m}^{c}=\frac{1}{P}\;\sum_{i,\;j}\frac{\partial z^{c}}{\partial A_{i,\;j}^{m}}$$ (8)

Here $\frac{\partial z^{c}}{\partial A^{m}}$ is the differentiable result of $z^{c}$ for a target class *c* with respect to the activation map *A*^*m*^ of a CONV layer.

## 4 Experiments and results analysis

### 4.1 Experiment environment

This section briefly discusses the experimental environment for the DCNN model for pneumonia prediction. In this experiment, we implemented the Keras library to establish an environment between DNN (deep neural network) and Python language. Table 5 lists the experimental setting and resources. The Flower FL model, as proposed in [54], serves as the inspiration for our proposed FL model.

**Table 5:** Environmental settings of the proposed system.

Resources	Details
GPU	Tesla K80
Platform	Google Colab
RAM	64 GB
CPU	Intel Core i5-12600K @ 3700 MHz

### 4.2 Performance Indicators

The classification accuracy, precision, F1-score, recall, and ROC curve are the evaluation metrics for the pneumonia prediction on CXR images. The scores of TP (true positive), FP (false positive), TN (true negative), and FN (false negative) are considered to calculate all these metrics utilizing the below equations 9–12.


$$Accuracy\;\left(Acc\right)=\;\frac{TP+TN}{TP+TN+FP+FN}$$ (9)

$$Precision\;\left(Pre\right)=\;\frac{TP}{TP+FP}$$ (10)

$$Recall\;\left(Rec\right)=\;\frac{TP}{TP+FN}$$ (11)

$$F1\;score\;\left(F1\right)=2*\frac{PRE*REC}{PRE+REC}$$ (12)

### 4.3 Result analysis

**Fig 6:**
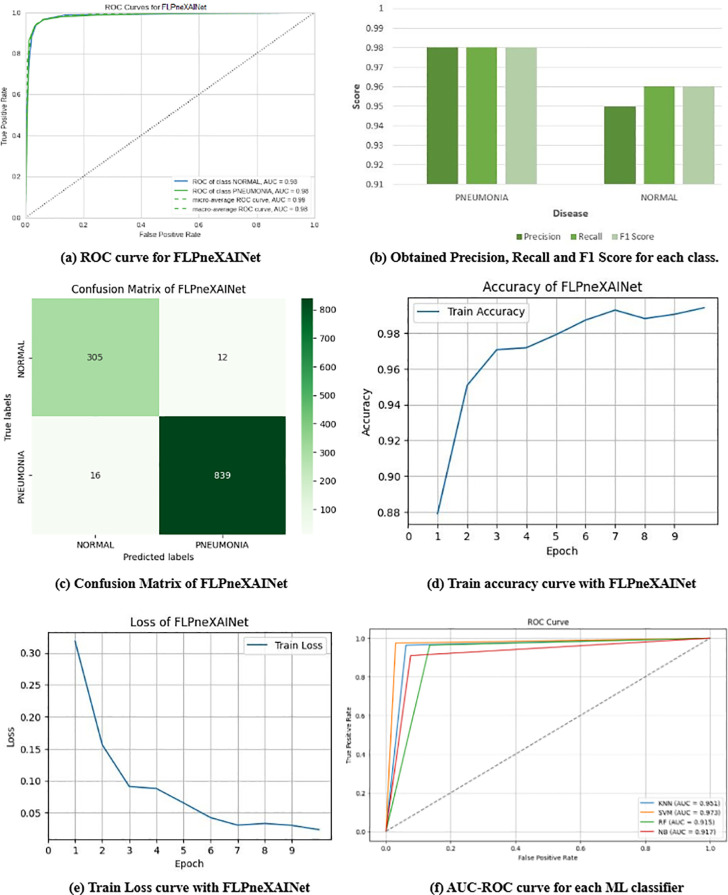
Performance indicators of proposed FLPneXAINet model.

We used three pre-trained DL models (MobileNet, VGG16, and NASNetMobile) for pneumonia prediction in this experiment. We also trained four ensemble DL (EDL) models to achieve better results. Fig 6 shows the performance indicators of the proposed FLPneXAINet framework. Fig 6(a) illustrates the ROC-AUC curve for two classes (pneumonia and normal), while Fig 6(b) illustrates the attained precision, F1-score, and recall scores. Fig 6(c)–6(e), respectively, demonstrate the CM (confusion matrix), training accuracy curve vs. epochs, and training loss curve vs. epochs. Finally, Fig 6(f) demonstrates the AUC-ROC curve of each ML classifier for predicting pneumonia.

Table 6 shows the predicted outcomes of both traditional and ensemble DL models for pneumonia prediction on the CXR data. Table 6 demonstrates that the EDL top-2 model (MobileNet + VGG16) achieves the highest accuracy (95.82%) among both traditional and ensemble DL models. However, we have also applied three feature optimizers (i.e., ANOVA, RF, and RFE) separately with this EDL-top2 model to get better prediction results. Table 7 displays the prediction outcomes of the EDL top-2 model with three feature optimizers. From Table 7, we clearly see that among these three optimizers, the EDL top-2 model with the RF optimizer exhibited improved accuracy (97.36%) than the single EDL top-2 model. However, training these traditional and ensemble DL models in an FL environment can improve the prediction results. In light of this, we evaluated the performance of three traditional DL models and the customized EDLNet in the FL environment. Table 8 shows the prediction results of different traditional DL models and EDLNet, with and without FL environments. Table 8 clearly demonstrates that EDLNet achieves the highest accuracy (97.35%) among the other models when trained and evaluated using GAN-augmented data in the FL environment. But, the accuracy of this network decreased by 1.79% when trained without GAN-augmented data and outside the FL environment. However, the VGG16 network exhibits the highest accuracy (97.35%) of the other models when trained without GAN-augmented data and outside the FL environment. We utilized four entities (clients), all having diverse datasets.

**Table 6:** Results of traditional and ensemble DL models of pneumonia prediction on CXR dataset.

Classifier	CNN Feature Extractors	Extracted feature	Acc (%)	Pre (%)	Rec (%)	F1 (%)	AUC (%)
SVM	VGG16	512	97.35	97.96	98.32	98.13	96.67
	NASNetMobile	1056	92.58	94.50	95.07	94.78	90.79
	MobileNet	1024	94.28	95.48	96.51	95.99	92.68
	VGG16 + NASNetMobile	1568	95.65	96.76	97.11	96.94	94.60
	NASNetMobile + MobileNet	2080	94.88	95.62	97.23	96.42	93.19
	MobileNet + VGG16 (**EDLNet**)	1536	95.82	96.44	97.71	97.07	94.46
	MobileNet+NASNetMobile+VGG16	2592	95.05	96.18	96.87	96.52	93.74

**Table 7:** Performance comparison of pneumonia prediction using several feature optimization approaches (i.e., RF, RFE and ANOVA).

Feature Extractor	Feature Selectors	Selected feature set	Classifiers	Acc (%)	Pre (%)	Rec (%)	F1 (%)
VGG16 + MobileNet (**optimized EDLNet**)	ANOVA	512	KNN	95.90	97.46	96.75	97.10
	RF	606		95.65	97.45	96.39	96.92
	RFE	784		94.28	95.91	96.03	95.97
	ANOVA	512	SVM	96.84	97.95	97.59	97.77
	RF	606		97.36	98.78	97.47	98.12
	RFE	784		96.59	97.25	97.95	97.60
	ANOVA	512	RF	93.26	94.34	96.27	95.30
	RF	606		93.52	94.57	96.39	95.47
	RFE	784		93.26	93.72	96.99	95.33
	ANOVA	512	NB	90.02	95.77	89.89	92.74
	RF	606		91.38	96.68	90.98	93.74
	RFE	784		88.57	98.07	85.56	91.39

**Table 8:** Performance comparison without and with FL environment.

	Without GAN and FL		With GAN and FL				
Model	Clients	Acc (%)	Clients	Acc (%)	Pre (%)	Rec (%)	F1 (%)
VGG16	–	97.35	4	97.44	97.14	99.42	98.23
MobileNet		94.28		97.44	97.91	98.60	98.25
NASNetMobile		92.58		96.50	97.88	97.31	97.60
VGG16 + MobileNet (**FLPneXAINet**)		95.82		97.61	98.59	98.13	98.36

### 4.4 XAI result analysis

**Fig 7:**
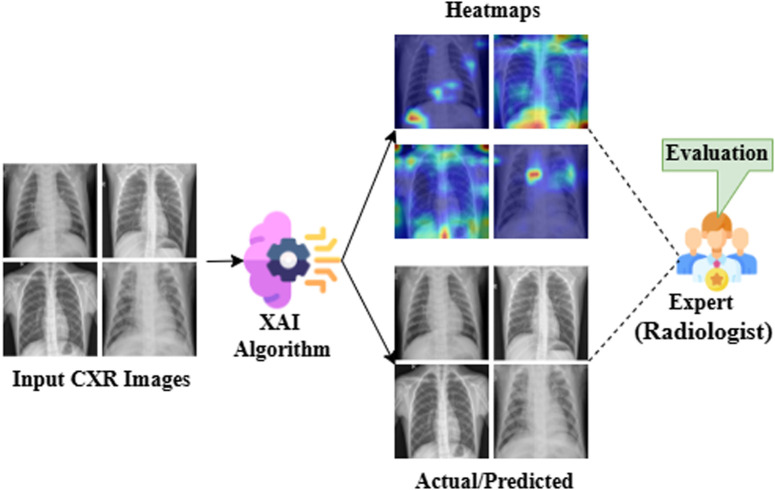
An example shows how XAI algorithm helps an expert to make a decision.

Fig 7 shows how the XAI algorithm helps an expert to make a decision. The GradCAM algorithm receives the predicted images generated by the proposed model as input. The GradCAM algorithm makes a heatmap result for each predicted image. Then a radiologist evaluates the predicted results with the generated heatmap. Thus, the XAI system helps a radiologist to make a decision based on the generated heatmap.

**Fig 8:**
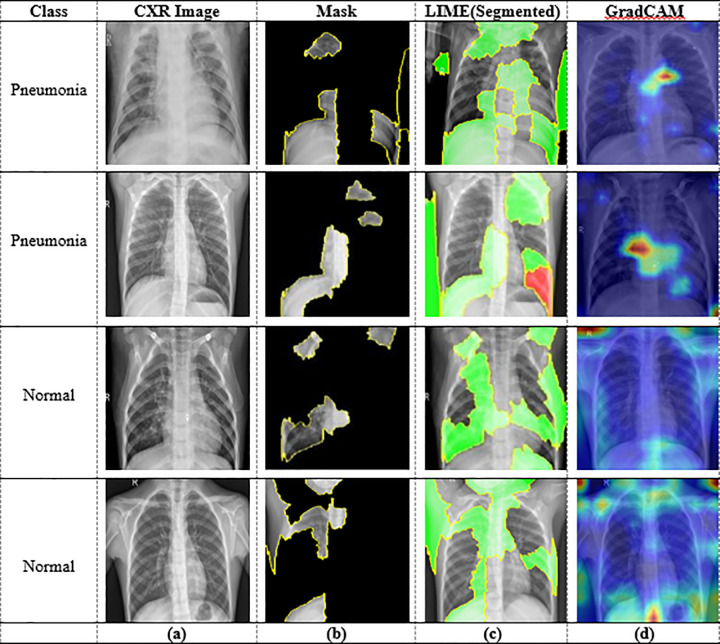
XAI result analysis by Grad-CAM and LIME: (a) input CXR image, (b) generated mask using LIME, (c) image segmentation using LIME, and (d) generated haetmap using Grad-CAM.

Fig 8 shows the generated results for predicted CXR images using LIME and GradCAM XAI algorithms to explain the predicted results of the proposed model: (a) input CXR image, (b) generated mask using LIME, (c) image segmentation using LIME, and (d) generated haetmap using GradCAM. In Fig 8(b), the LIME algorithm generates new samples by masking several regions of the image. Fig 8(c) also shows that the LIME algorithm segments the predicted images into small regions for local approximations and interpretation, and each region is interconnected with another in the same color. On the other hand, Fig 8(d) shows which areas of the CXR samples had the most impact on the prediction of the proposed model. The Grad-CAM XAI approach is used to highlight these most influential areas that are integrated into the proposed model during the model evaluation. In Fig 8(d), the red region indicates higher chances of pneumonia, while the yellow area indicates lower chances of pneumonia. Thus, by highlighting the most influential regions in the pneumonia detection system, this algorithm helps clinicians understand the model’s decision-making process.

### 4.5 Computational complexity analysis

For complexity analysis, assume that the number of CXR data is n, the number of total retrieved characteristics is m, and the number of selected features from the fused set is g. In EDLNet with feature optimization, Optimized EDLNet has two steps: extraction of DL features and selection of the most important features. In feature extraction, extracting the features using a pre-trained DCNN model takes O(nm) time. When selecting important features, using multiple feature selectors takes O(ng) time. As g<=m, the overall time complexity of the optimized EDLNet is O(nm)+O(ng)=O(nm).

### 4.6 Discussion

This work suggests a method for predicting pneumonia that combines FL with the CycleGAN image enhancement technique. This methodology ensures data privacy while classifying CXR images as either pneumonia or normal across several clients. Implementing this system reduces the need for image data sharing, thereby eliminating concerns related to information leakage. Our method has the potential to improve medical image analysis with higher degree accuracy, especially in the medical sector. We assure data confidentiality by confirming patient information with the local server [55]. To conduct this experiment, an EDL model, consisting of MobileNet and VGG16, utilized for evaluating and computing the global network’s parameters.

In the rapidly developing medical industry, various authors have introduced DL-based approaches for predicting pneumonia from the CXR dataset in their published articles. However, in all papers from [56–64], authors differentiated pneumonia from normal chest x-ray images using their methods. In this section, we discuss the novelty of our suggested framework based on their published articles.

Our suggested framework exhibits better precision and accuracy compared to the above-mentioned methods, which reflects that it can accurately detect pneumonia samples. Significantly, a high recall of 98.13% highlighted the framework’s capacity to reduce false negatives, which is an important trait in medical industries. The framework’s superior recall complemented a precision of 98.59%, slightly less than from [57], and the F1-score successfully balanced both metrics. We achieved our framework’s accuracy within only ten training epochs, demonstrating its practical applicability and the effectiveness of the training approach. Similarly, while the proposed framework reached the highest precision in [57], it did so at the expense of fewer recalls, which could outcome in a greater number of false negatives. In [58], the proposed framework demonstrated a balanced F1-score and precision, but failed to reach near the recall score of our suggested framework. In [58], the proposed framework presented comparable results across all performance indicators, reflecting a roughly balanced framework, but the overall performance was much lower than our framework. In [60], the proposed framework showed a relatively higher level of precision, but its overall performance was not as high as our suggested framework.

In summary, the suggested framework not only reveals superior performance in recall and accuracy but also significantly improves these metrics over a few training epochs. Its effectiveness and high performance metrics make it a strong candidate for automatic pneumonia detection from CXR images in the medical industry. The evaluation metrics of the above-mentioned frameworks and our suggested framework are listed in Table 9.

**Table 9:** Comparative results for the SOTA approach on test cases of the CXR dataset. The top results for each articles are indicated in bold. Here, ACC stands for accuracy, PRE stands for precision, REC stands for recall and F1 stands for F1 Score.

Papers	Dataset Size	Method	ACC (%)	PRE (%)	REC (%)	F1 (%)
Sharma *et al.* [56] (2023)	6436	VGG16 + NN	92.15	93.08	94.28	93.70
Bhatt *et al.* [57] (2023)	5863	Ensemble CNN	84.12	**99.23**	80.04	88.56
Goyal *et al.* [58] (2023)	5856	RNN-LSTM DL	94.31	95.41	88.89	92.03
Mabrouk *et al.* [59] (2023)	5856	MobileNetV2 + Vision Transformer+ DenseNet169	93.91	92.99	93.96	93.43
Wang *et al.* [60] (2022)	5856	DenseNet + SE	92.80	96.20	92.60	94.30
An *et al.* [61] (2024)	5856	EfficientNetB0 + DenseNet121 + Attention Network	95.19	98.38	93.84	96.06
Ojewumi *et al.* [62] (2024)	5856	VGG16	94	91	95	93
Ren *et al.* [63] (2024)	2572	CheXMed	74.6	74.6	74	74.3
Ali *et al.* [64] (2024)	5856	EfficientNetV2L	94.02	94.40	97.24	95.80
**Proposed**	**8402**	**MobileNet + VGG16 + FL+ CycleGAN**	**97.61**	98.59	**98.13**	**98.36**

However, there are some limitations in our proposed framework, such as the fact that it ignores patients’ medical histories, experiential gaps, and other bodily symptoms in population data, and some human oversight is still necessary. On the other hand, this proposed approach only applicable for binary classification. In future, we have a plan to train and test our model on a multi-disciplinary dataset consists of covid19 and tuberculosis (TB) cases.

## 5. Conclusion and future scope

This paper presented a secure and effective system for pneumonia prediction that leverages Federated Learning (FL) and an Ensemble Deep Learning (EDL) approach “FLPneXAINet” using CXR images. By incorporating FL, we ensure that data confidentiality is maintained throughout the training process, keeping sensitive information secure. Given the limited availability of CXR images, we employed a CycleGAN network to augment the training data. These augmented images were then partitioned and assigned to different entities or clients, where local models were trained using various traditional and ensemble DL algorithms. Among these, the ensemble DL network EDLNet, which integrates MobileNet and VGG16 with an SVM classifier, demonstrated outstanding performance, achieving an accuracy of 95.82%, precision of 96.44%, recall of 97.71%, and an F1 score of 97.07%. Further enhancement was achieved using three feature optimization techniques: Random Forest (RF), Recursive Feature Elimination (RFE), and ANOVA. The EDLNet model optimized with the RF feature optimizer delivered the highest classification performance, with an accuracy of 97.36%, precision of 98.78%, recall of 98.47%, and an F1 score of 98.12%. To evaluate the effectiveness of the optimized EDLNet, we compared it with traditional DL algorithms within the FL environment, finding that the FL-EDLNet provided even better results. The FL-EDLNet achieved an accuracy of 97.61%, precision of 98.59%, recall of 98.13%, and an F1 score of 98.36%. This proposed approach is particularly effective in medical settings where datasets are limited, and data privacy is important. Future enhancements could involve integrating other data-secure platforms, such as blockchain, to further fortify the confidentiality of CXR images. Additionally, combining multi-modal CXR datasets with clinical data and exploring ensemble deep learning models from related medical fields could further improve the system performance.
